# Supplementary material for: Phosphorylation remodels the mitotic centrosome matrix to generate bipartite γ-tubulin complex docking sites
Source: bioRxiv. 2025 Nov 21:2025.11.20.689565. Preprint. [Version 1] doi: 10.1101/2025.11.20.689565 (PMC12667865; doi:10.1101/2025.11.20.689565)
Supplement: 1 [file NIHPP2025.11.20.689565v1-supplement-1.pdf]

bioRxiv preprint doi: https://doi.org/10.1101/2025.11.20.689565; this version posted November 21, 2025. The copyright holder for this preprint (which was not certified by peer review) is the author/funder, who has granted bioRxiv a license to display the preprint in perpetuity. It is made available under aCC-BY 4.0 International license.

Supplementary Figures and Tables

Figure S1

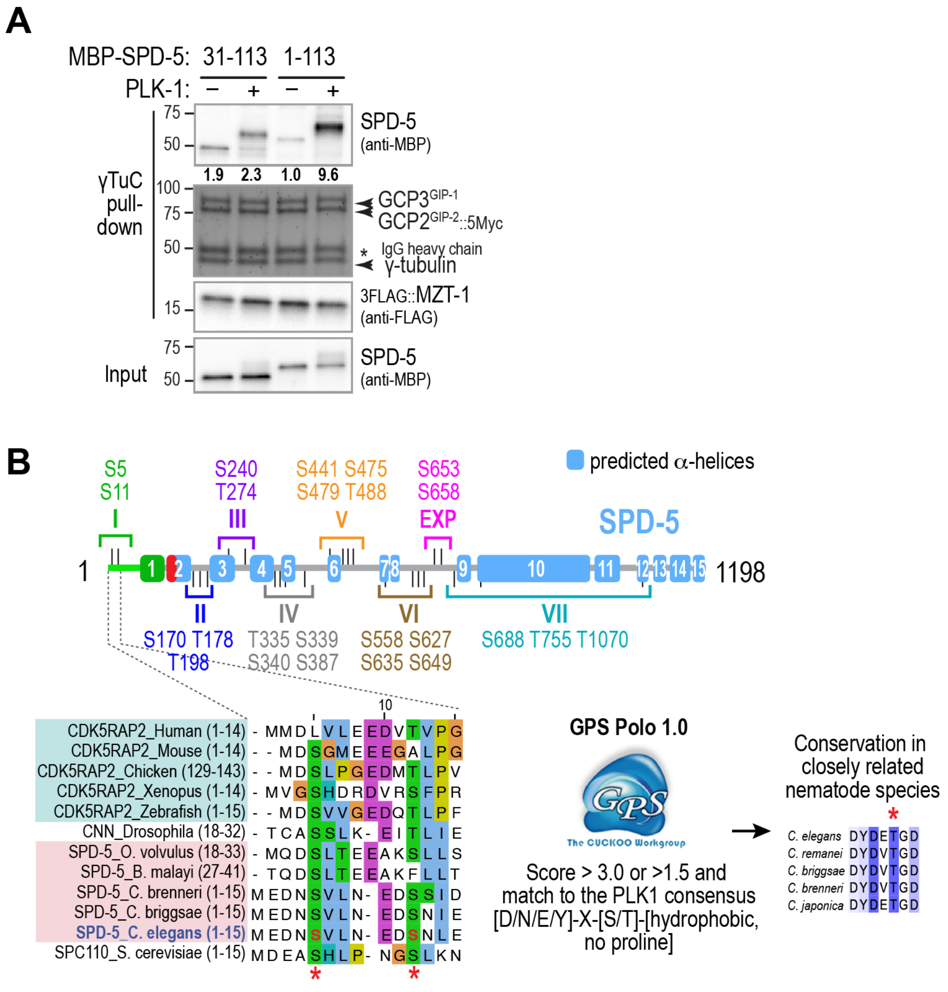

Fig. S1. Putative PLK1 target sites in PRGB1 and their sequence alignment. (**A**) Binding assays, conducted as outlined in Fig. 1C, with γTuC-coated beads and MBP-6His-HA (M6HH)-tagged SPD-5 fragments, preincubated with or without PLK1 as indicated. SPD-5 and MZT-1 were analyzed by immunoblotting using the indicated antibodies; γ-tubulin, GCP2^{GIP-2}, and GCP3^{GIP-1} were detected by Coomassie staining. Numbers below the SPD-5 fragment bands indicate band intensity relative to SPD-5 1-113 in the absence of PLK1 phosphorylation. Asterisk indicates the IgG heavy chain of the anti-Myc antibody used for the Myc IP. The blot for SPD-5 1-113 is the same as that used in Fig. 1E. (**B**) Top: Schematic depicting candidate PLK1 sites identified using the method shown in the lower right that were mutated in the indicated regional clusters in a prior study that identified cluster II as causing penetrant embryonic lethality (*32*). Bottom Left: Alignment of the indicated sequences from the N-termini of CDK5RAP2 family proteins across species, highlighting potential conservation of the S5 and S11 sites. Phosphorylation sites in alignments are marked with red asterisks.

bioRxiv preprint doi: https://doi.org/10.1101/2025.11.20.689565; this version posted November 21, 2025. The copyright holder for this preprint (which was not certified by peer review) is the author/funder, who has granted bioRxiv a license to display the preprint in perpetuity. It is made available under aCC-BY 4.0 International license.

Figure S2

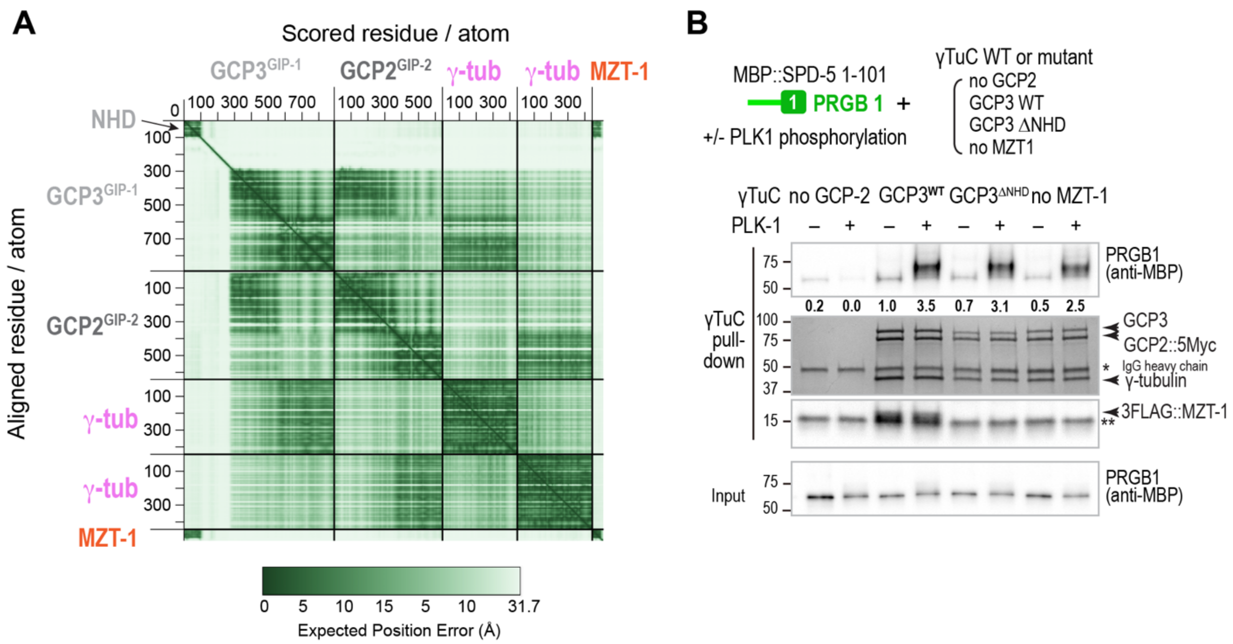

Fig. S2. MZT-1 is dispensable for PRGB1 binding to γTuC. (A) Predicted Aligned Error (PAE) plot generated using PAEViewer (*66*) for the *C. elegans* γTuC model in Fig. 4A. The MZT-1–GCP3^{GIP-1} NHD module is separated by a disordered linker and is not positioned relative to the core heterotetrameric complex. **(B)** Expanded version of the western blots/coomassie gel shown in *Fig. 4C* that includes controls in which the plasmids encoding GCP2 which has the Myc tag for the IP (*left two lanes*) or MZT-1 (right two lanes) were omitted during γTuC assembly. Like deletion of the GCP3^{GIP-1} NHD, assembling the γTuC without MZT-1 also does not impact its PLK1-dependent binding to PRGB1. Beads prepared in with the indicated γTuC components were incubated with MBP-6His-HA-tagged SPD-5 aa 1-101 after preincubation with or without PLK1 as indicated. SPD-5 and MZT-1 were analyzed by immunoblotting using the indicated antibodies; γ-tubulin, GCP2^{GIP-2}, and GCP3^{GIP-1} were detected by Coomassie staining. Numbers below the PRGB1 bands indicate band intensity relative to PRGB1 pulled down by γTuC assembled in the presence of WT GCP3 in the absence of PLK1 phosphorylation. Single asterisk indicates the IgG heavy chain of the anti-Myc antibody used for the Myc IP. Double asterisk marks the location of a non-specific band.

bioRxiv preprint doi: https://doi.org/10.1101/2025.11.20.689565; this version posted November 21, 2025. The copyright holder for this preprint (which was not certified by peer review) is the author/funder, who has granted bioRxiv a license to display the preprint in perpetuity. It is made available under aCC-BY 4.0 International license.

Figure S3

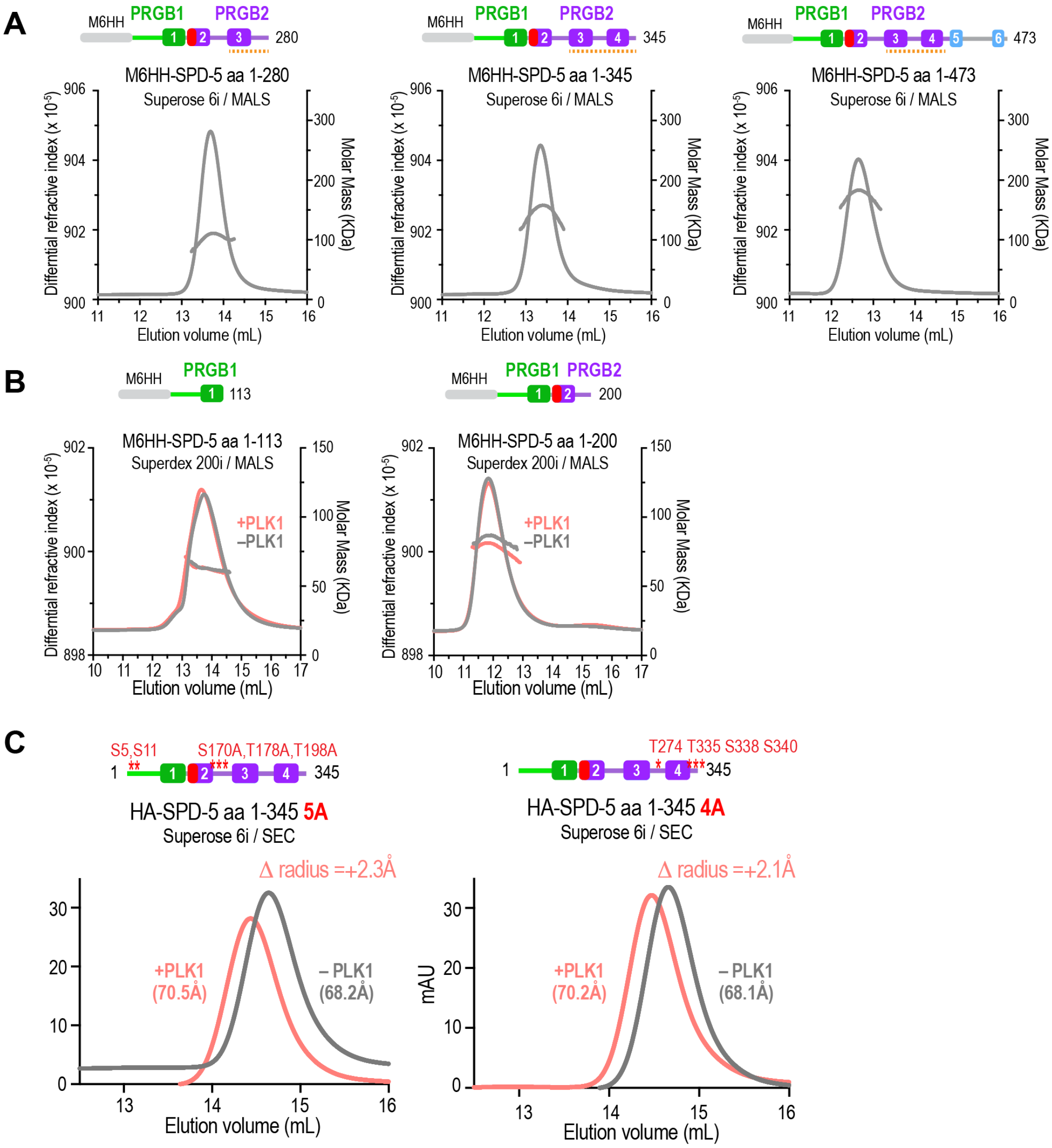

bioRxiv preprint doi: https://doi.org/10.1101/2025.11.20.689565; this version posted November 21, 2025. The copyright holder for this preprint (which was not certified by peer review) is the author/funder, who has granted bioRxiv a license to display the preprint in perpetuity. It is made available under aCC-BY 4.0 International license.

Fig. S3. The SPD-5 N-terminus undergoes a PLK1-stimulated conformational change as the result of distributed phosphorylation across the N-terminus. (A) SEC-MALS data for MBP-6His-HA-tagged (M6HH)-tagged SPD-5 aa 1-280 and SPD-5 aa 1-345 used to determine the values reported in Fig. 5A. **(B)** SEC-MALS data for MBP-6His-HA-tagged (M6HH)-tagged SPD-5 aa 1-113 and SPD-5 aa 1-200 before and after PLK1 phosphorylation used to determine the values in Fig. 5A. The two fragments are monomeric both in the presence and absence of PLK1 phosphorylation and do not exhibit a phosphorylation-dependent shift in elution volume. Orange dashed line marks the region required for dimerization. **(C)** Size exclusion chromatography (SEC) analysis of HA-tagged SPD-5 aa 1-345 with the indicated sets of predicted PLK1 sites mutated to alanine after preincubation with or without PLK1 as indicated. The hydrodynamic radii of the SPD-5 fragments were calculated based on standard proteins and are shown in parentheses and the change in radius induced by phosphorylation is in the upper right corner of each graph.

bioRxiv preprint doi: https://doi.org/10.1101/2025.11.20.689565; this version posted November 21, 2025. The copyright holder for this preprint (which was not certified by peer review) is the author/funder, who has granted bioRxiv a license to display the preprint in perpetuity. It is made available under aCC-BY 4.0 International license.

Table S1. ***C. elegans*** **strains used in this study.**

Strain #	Genotype	Figure
OD4412	ltSi569[oxTi185; pOD1110/pSW008; CEOP3608 TBG-1::mCherry; cb-unc-119(+)]I; ltSi1216 [pOD1021/pVV103; Pspd-2::GFP::SPD-5 reencoded; cb-unc-119(+)]II; unc-119(ed3) III	2A-C, 3B,
MOW11	ltSi569[oxTi185; pOD1110/pSW008; CEOP3608 TBG-1::mCherry; cb-unc-119(+)]I; ltSi1967 [pMO1326; Pspd-2::GFP::SPD-5 reencoded (deletion aa61-93)::spd-5 3'UTR; cb-unc-119(+)]II	2A
OD5196	ltSi569[oxTi185; pOD1110/pSW008; CEOP3608 TBG-1::mCherry; cb-unc-119(+)]I; ltSi1532 [pMO91; Pspd-2::gfp::spd-5 S5A/S11A::spd-5 3'UTR; cb-unc119(+)]II; unc-119(ed3) III	2B
OD5220	ltSi569[oxTi185; pOD1110/pSW008; CEOP3608 TBG-1::mCherry; cb-unc-119(+)]I; ltSi1647 [pMO253; Pspd-2::GFP::SPD-5 aa61-1198 reencoded; cb-unc-119(+)]II; unc-119(ed3) III	2B
MOW09	ltSi569[oxTi185; pOD1110/pSW008; CEOP3608 TBG-1::mCherry; cb-unc-119(+)]I; ltSi1704 [pMO269; Pspd-2::GFP::SPD-5 aa264-1198 reencoded; cb-unc-119(+)]II; unc-119(ed3) III	2C
MOW21	ltSi569[oxTi185; pOD1110/pSW008; CEOP3608 TBG-1::mCherry; cb-unc-119(+)]I; ltSi2010 [pMO1503; Pspd-2::GFP::SPD-5 reencoded (deletion aa135-180)::spd-5 3'UTR; cb-unc-119(+)]II; unc-119(ed3)III (#18)	3B
OD4211	ltSi1216[pOD1021/pVV103; Pspd-2::GFP::SPD-5 reencoded; cb-unc-119(+)]II; unc-119(ed3) III	2D
OD5160	ltSi1641 [pMO252; Pspd-2::GFP::SPD-5 aa114-1198 reencoded; cb-unc-119(+)]II; unc-119(ed3) III	2D
OD5262	ltSi1704[pMO269; Pspd-2::GFP::SPD-5 aa264-1198 reencoded; cb-unc-119(+)]II; unc-119(ed3) III	2D

bioRxiv preprint doi: https://doi.org/10.1101/2025.11.20.689565; this version posted November 21, 2025. The copyright holder for this preprint (which was not certified by peer review) is the author/funder, who has granted bioRxiv a license to display the preprint in perpetuity. It is made available under aCC-BY 4.0 International license.

Table S2. Oligos used for dsRNA production.

**Gene**	**Oligonucleotide 1 (5' → 3')**	**Oligonucleotide 2 (5' → 3')**	**Template**	**Concentration (mg/mL)**
spd-5 (F56A3.4)	TAATACGACTCACTATAGGTGGAATTGTCCGCTACTGAT	AATTAACCCTCACTAAAGGTGTATTCAACGAGTGCCTGA	N2 genomic DNA	1.3 - 1.4

bioRxiv preprint doi: https://doi.org/10.1101/2025.11.20.689565; this version posted November 21, 2025. The copyright holder for this preprint (which was not certified by peer review) is the author/funder, who has granted bioRxiv a license to display the preprint in perpetuity. It is made available under aCC-BY 4.0 International license.

Table S3. Plasmids used in this study.

plasmid #	Description	Bacterial selection
**pMO97**	p3XFLAG-CMV-7.1	Ampicillin
**pMO98**	SP254_CS2P_mtc2	Ampicillin
**pMO103**	pCMV-TBG-1 (*C. elegans* γ-tubulin)	Ampicillin
**pMO106**	pCMV-GIP-2-5Myc	Ampicillin
**pMO113**	pCMV-GIP-1	Ampicillin
**pMO135**	pCMV-3FLAG-MZT-1	Ampicillin
**pMO133**	pGEX-6P-1-GST-SPD-5 aa1-473	Ampicillin
**pMO193**	pGEX-6P-1-GST-SPD-5 aa181-473	Ampicillin
**pMO205**	pGEX-6P-1-GST-SPD-5 aa101-473	Ampicillin
**pMO208**	pGEX-6P-1-GST-SPD-5 aa135-473	Ampicillin
**pMO245**	pGEX-6P-1-GST-HA-SPD-5 aa1-270	Ampicillin
**pMO247**	pGEX-6P-1-GST-HA-SPD-5 aa1-345	Ampicillin
**pMO233**	pGEX-6P-1-GST-HA-SPD-5 aa1-473	Ampicillin
**pMO258**	pMAL-His6-TEV-HA-SPD-5 aa1-113	Ampicillin
**pMO260**	pMAL-His6-TEV-HA-SPD-5 aa1-200	Ampicillin
**pMO263**	pMAL-His6-TEV-HA-SPD-5 aa1-280	Ampicillin
**pMO264**	pMAL-His6-TEV-HA-SPD-5 aa59-113	Ampicillin
**pMO285**	pMAL-His6-TEV-HA-SPD-5 aa1-345	Ampicillin
**pMO287**	pMAL-His6-TEV-HA-SPD-5 aa1-137	Ampicillin
**pMO288**	pMAL-His6-TEV-HA-SPD-5 aa1-170	Ampicillin
**pMO290**	pGEX-6P-1-GST-HA-SPD-5 aa1-113 S5A S11A	Ampicillin
**pMO292**	pMAL-His6-TEV-HA-SPD-5 aa1-473	Ampicillin
**pMO293**	pMAL-His6-TEV-HA-SPD-5 aa31-113	Ampicillin
**pMO302**	pMAL-His6-TEV-HA-SPD-5 aa1-101	Ampicillin
**pMO303**	pMAL-His6-TEV-HA-SPD-5 aa1-60	Ampicillin
**pMO306**	pGEX-6P-1-GST-HA-SPD-5 aa1-345 S5A S11A S170A T178A T198A	Ampicillin
**pMO307**	pGEX-6P-1-GST-HA-SPD-5 aa1-345 T274A T335A S339A S340A	Ampicillin

bioRxiv preprint doi: https://doi.org/10.1101/2025.11.20.689565; this version posted November 21, 2025. The copyright holder for this preprint (which was not certified by peer review) is the author/funder, who has granted bioRxiv a license to display the preprint in perpetuity. It is made available under aCC-BY 4.0 International license.

**pMO308**	pGEX-6P-1-GST-HA-SPD-5 aa1-345 S5A S11A S170A T178A T198A T274A T335A S339A S340A	Ampicillin
**pMO1323**	pCMV-GIP-1 delta NHD	Ampicillin
**pMO1358**	pMAL-His6-TEV-HA-SPD-5 aa135-200	Ampicillin
**pMO1360**	pMAL-His6-TEV-HA-SPD-5 aa135-280	Ampicillin
**pMO1362**	pMAL-His6-TEV-HA-SPD-5 aa135-345	Ampicillin
**pMO1368**	pGEX-6P-1-GST-SPD-5 aa135-473 S170A T178A T198A	Ampicillin
**pMO1370**	pGEX-6P-1-GST-SPD-5 aa135-473 T274A T335A S339A S340A	Ampicillin
**pMO1372**	pGEX-6P-1-GST-SPD-5 aa135-473 S170A T178A T198A T274A T335A S339A S340A	Ampicillin
**pMO1389**	pHalo-His6-TEV-HA-SPD-5 aa135-200	Ampicillin
**pMO1401**	pHalo-His6-TEV-HA-SPD-5 aa135-345	Ampicillin
**pMO1402**	pHalo-His6-TEV-HA-SPD-5 aa200-345	Ampicillin
**pMO1404**	pHalo-His6-TEV-HA-SPD-5 aa260-345	Ampicillin
**pMO1670**	pMAL-His6-TEV-HA-SPD-5 aa1-263	Ampicillin
